# Sociodemographic and Medical Determinants of Quality of Life in Long-Term Childhood Acute Lymphoblastic Leukemia Survivors Enrolled in EORTC CLG Studies

**DOI:** 10.3390/cancers14010152

**Published:** 2021-12-29

**Authors:** Charlotte Sleurs, Jammbe Musoro, Ali Rowsell, Michal Kicinski, Stefan Suciu, Sofia Chantziara, Corneel Coens, Madeline Pe, Pierre Missotten, Els Vandecruys, Anne Uyttebroeck, Marie-Françoise Dresse, Claire Pluchart, Alina Ferster, Claire Freycon, Jutte van der Werff ten Bosch, Pierre-Simon Rohrlich, Yves Benoit, Anne-Sophie Darlington, Caroline Piette

**Affiliations:** 1 Department of Pediatric Hemato-Oncology, University Hospital Leuven, 3000 Leuven, Belgium; charlotte.sleurs@kuleuven.be (C.S.); anne.uyttebroeck@uzleuven.be (A.U.); 2 European Organisation for Research and Treatment of Cancer (EORTC), 1200 Brussels, Belgium; jammbe.musoro@eortc.org (J.M.); michal.kicinski@eortc.org (M.K.); stefan.suciu@eortc.org (S.S.); corneel.coens@eortc.org (C.C.); madeline.pe@eortc.org (M.P.); 3 School of Health Sciences, University of Southampton, Southampton SO17 1BJ, UK; A.C.Rowsell@soton.ac.uk (A.R.); sofiacha11@googlemail.com (S.C.); a.darlington@soton.ac.uk (A.-S.D.); 4 Unité de Psychologie de la Sénescence, Université de Liège, 4000 Liege, Belgium; pmissotten@uliege.be; 5 Department of Pediatric Hematology-Oncology, Ghent University Hospital, 9000 Ghent, Belgium; els.vandecruys@uzgent.be (E.V.); yves.benoit@uzgent.be (Y.B.); 6 Division of Haematology-Oncology, Department of Paediatrics, University Hospital Liège, University of Liège, 4000 Liege, Belgium; mf.dresse@chuliege.be; 7 Department of Pediatric Haematology and Oncology, CHU Reims, 51100 Reims, France; cpluchart@chu-reims.fr; 8 Department of Hemato-Oncology, HUDERF (ULB), 1020 Brussels, Belgium; alina.ferster@huderf.be; 9 Department of Pediatric Hematology-Oncology, CHU Grenoble, 38700 Grenoble, France; cfreycon@chu-grenoble.fr; 10 Department of Pediatrics, UZ Brussel, 1090 Brussels, Belgium; jutte.VanderWerffTenBosch@uzbrussel.be; 11 Pediatric Oncology, CHU Nice, 06200 Nice, France; rohrlich.ps@chu-nice.fr

**Keywords:** acute lymphoblastic leukemia, quality of life, long-term survivorship

## Abstract

**Simple Summary:**

Long-term quality of life and its potential risk factors in childhood acute lymphoblastic leukemia (ALL) patients remain uncertain. In this cross-sectional study, we investigated daily life quality and life challenges in adult survivors of ALL using multiple self-report questionnaires. Furthermore, risk factors, including gender, age at diagnosis, relapse/second neoplasm, risk group, and cranial radiotherapy, were explored in detail. Younger, female, and relapsed patients appeared to encounter more life challenges, while physical challenges occurred more often in relapsed and high-risk patients. More positive effects on socializing were found in the older patients compared to younger patients. This study provides important information for individual and specialized support.

**Abstract:**

Background: due to increasing survival rates in childhood acute lymphoblastic leukemia (ALL), the number of survivors has been expanding. A significant proportion of these survivors can experience long-term emotional and psychosocial problems. However, the exact risk factors remain inconclusive. We investigated potential risk factors for decreased daily life quality and life challenges in long-term childhood ALL survivors enrolled between 1971 and 1998 in EORTC studies. Methods: self-report questionnaires were collected from 186 survivors (109 females; mean age at diagnosis 5.62 years, range 0.2–14.7; median time since diagnosis of 20.5 years (12.9–41.6)), including the Short-Form Health Survey (SF-12) and Impact of Cancer-Childhood Survivors (IOC-CS). Multivariable linear regression models were used to assess the impact of gender, age at diagnosis, relapse/second neoplasm, National Cancer Institute (NCI) risk group and cranial radiotherapy on 2 subscales of the SF-12 (physical and mental health) and five subscales of the IOC-CS (life challenges, body and health, personal growth, thinking and memory problems and socializing). Results: mental component scores of SF-12 were not significantly associated with any risk factor. Physical component scores were lower in relapsed, irradiated and NCI high-risk patients. Regarding IOC-CS negative impact subscales, life challenges was more negatively impacted by cancer in female, younger (i.e., <6 years) and relapsed patients. Regarding the positive impact scales, personal growth was more positively impacted in relapsed patients, whereas body and health, and socializing, were less positively impacted in these patients, compared to non-relapsed patients. Socializing was more positively impacted in older patients (>6 years). Conclusions: this study demonstrates that long-term outcomes can be both adverse and positive, depending on the patient’s demographic and clinical characteristics. Younger, female, and relapsed patients might encounter more life challenges years after their disease, while physical challenges could occur more often in relapsed and high-risk patients. Finally, the positive effect on socializing in the older patients sheds new light on the importance of peer interactions for this subgroup. Specific individual challenges thus need specialized support for specific subgroups.

## 1. Introduction

Acute lymphoblastic leukemia (ALL) is the most common type of cancer in children and adolescents. Given that the 5-year survival rate of ALL reaches up to 90%, the number of survivors has exponentially expanded throughout recent decades. The quality of life (QoL) of these long-term survivors can be compromised by sequelae [1,2], unpredictable health situations [3], depressive mood, fatigue, or weakness secondary to the ALL and its treatments [4].

A significant proportion of patients experience these problems shortly after treatment (i.e., <1 year post-treatment) [4]. Although QoL is often observed to recover or improve throughout their development, some patients might continue to experience mental or physical difficulties into adulthood [5,6,7,8,9,10,11]. In a limited number of cases, such experiences can sometimes even lead to psychiatric problems such as obsessive-compulsive and oppositional defiant disorders in adolescents [12] or increased use of psychopharmacological agents (e.g., neuroleptics, tranquilizers or antidepressants) in young adults [13]. Given that leukemia is most often diagnosed around the age of 4 to 5 years old, the psychological development of the child can strongly be affected, which could in the longer run also affect the adult stage of life, during which survivors are confronted with new life challenges (e.g., sexuality, intimate relationships, job opportunities, household, etc.).

Given that QoL research in adult survivors remains limited, the question of much interest is which demographic and medical risk factors could determine the risk of patients developing long-term problems and decreased QoL.

So far, the impact of risk factors was mostly investigated in adolescent survivors, showing some effects of treatment intensity as well as demographic features. For instance, cranial radiotherapy (CRT) was concluded to be a possible risk factor for poorer QoL in survivorship studies [14,15]. However, its impact can depend on gender (e.g., stronger in females [16]), age or other factors. In addition, chemotherapy [17] and allogeneic hematopoietic stem cell transplantation can also lead to long-term adverse sequelae such as pain [10], fatigue [18], anxiety and depression [10,19], while corticosteroids are mostly associated with worse QoL [17,20] and emotional and behavioral problems [21] after shorter intervals. In addition to these treatment effects, patients who relapsed might be at increased risk for poorer health perception due to their increased number of late effects [22]. Moreover, patients in the “high risk” treatment groups might be more vulnerable for worse QoL outcomes [2,23]. Still, risk group definitions and risk-adjusted therapeutic strategies depend on the specified treatment protocol, which could explain, at least to some extent, a certain variability across studies [24]. From a sociodemographic point of view, education, being married, being employed [25], or higher financial income can be protective factors [26], and male survivors might have less emotional problems compared to females [1,13]. 

Although these former studies already highlighted some potential risk factors for altered QoL in childhood ALL patients, differences are encountered between self- and parent-reported QoL [12,27]. More specifically, parents could tend to report more problems in female and in older patients [2,6,16,28], or more mental symptoms in general than the survivors themselves [29]. Furthermore, most studies included current ALL patients or the assessments were collected only shortly (e.g., <1 y) after treatment. In other words, patients were still at the same developmental stage of life as when they were diagnosed, when questions regarding their QoL were posed. However, as patients develop and become (emerging) adults, they live in a different stage of life and are then faced with new challenges (compared to their life stage at diagnosis). In adulthood, the QoL outcomes and associations with risk factors can be very different in long-term adult survivors from the abovementioned studies. 

Given that associations between individual risk factors at diagnosis and long-term outcomes in adulthood are currently insufficiently investigated, we investigated the QoL and daily life functioning of adult survivors of childhood ALL, more than 10 years post-diagnosis, using multiple questionnaires in a large survivorship cohort. 

## 2. Methods

### 2.1. Design

Adult survivors (≥18 years) who were diagnosed with ALL during childhood (<18 years at diagnosis) and registered in the European Organisation for Research and Treatment of Cancer (EORTC) Children Leukaemia Group (CLG) trials 58741 (1971–1978), 58831/2 (1983–1989) and 58881 (1989–1998) were eligible. Details regarding the risk-group stratification and the treatment protocols were described earlier [30,31] and provided in Figure S1. Follow-up data, including QoL data, were collected from 24 institutions (10 in Belgium and 14 in France). 

This study is part of a larger EORTC CLG study (58 Late Adverse Effects (LAE)), which was registered on ClinicalTrials.gov (NCT01298388) and approved by the Ethical Committees of the participating institutions. At the time of the enrolment in studies 58741, 58831/2 and 58881, informed consent was sought according to local practice of each participating center and in accordance with the Declaration of Helsinki. The EORTC study 58LAE was approved by the Ethical Committees of the participating institutions and informed consent was obtained from all patients, in accordance with the applicable national legislation.

### 2.2. Measures

The questionnaires “Short-Form Health Survey (SF-12)” and “Impact of Cancer-Childhood Survivors (IOC-CS)” were either sent by post or given to the patient during their visit at the hospital. All questionnaires were completed by the participants between 2012 and 2017, and collected at the EORTC HQ. 

The SF-12 comprises eight main subscales (general health, physical functioning, bodily pain, role-physical, vitality, social functioning, role-emotional, and mental health), which are further summarized into physical component summary (PCS) and a mental component summary (MCS) scores. PCS and MCS were scored based the scoring method by Ware et al. [32] with scores ranging from 0 (worst possible health) to 100 (optimal health).

The IOC-CS consists of 45 items summarized into eight subscales: five positive impact subscales (body and health, personal growth, talking with parents and health literacy and socializing) and three negative impact scales (life challenges, thinking and memory problems and financial problems). These subscales were scored, according to published guidelines [33,34], by calculating the mean of all items (on an ordinal scale, where 1 indicated no impact at all, and 5 indicated a large impact) within their corresponding subscales. For positive impact scales, a higher mean score indicates a larger positive impact, while for the IOC-CS negative impact scales, a higher mean score indicates a larger negative impact. Based on the literature and expert opinion, specific outcomes of interest were preselected (Table S1). Consequently, for the purpose of this study, we analyzed five subscales: three positive impact subscales (body and health and personal growth and socializing), and two negative impact subscales (life challenges, thinking and memory problems). 

### 2.3. Definitions

Second neoplasm (SN) was defined as any malignancy distinct from the initial ALL diagnosis, whatever the remission status of the patient. Benign tumors were not considered as SN, except for tumors of the central nervous system.

The National Cancer Institute (NCI) risk group was defined according to the age and the white blood cells (WBC) count at diagnosis [35].

### 2.4. Data Analyses

Given the limited sample size, statistical models were constructed as sparse as possible. Hence, predictors of pre-specified QoL subscales were gender, age at diagnosis, relapse/SN, NCI risk group, and CRT. For each predictor of interest, a separate linear regression model was fitted for each of the corresponding QoL subscale. Table S1 summarizes the QoL subscale outcomes and sets of predictors that were used for the various models. This selection of the QoL scales and adequate covariates of interest (Table S1) as well as potential confounders was based on clinical expert opinion and findings from the literature. Both the unadjusted (based on a univariate linear regression) and adjusted (based on a multivariable linear regression, including predefined covariates) effects were estimated. A *p*-value ≤ 0.05 was used as a screening measure to get an indication of statistical strength (no adjustment for multiple comparisons was applied given that the models were predefined based on expert opinion). All statistical analyses were performed using Statistical Analysis System software [36]. 

## 3. Results 

### 3.1. Participants

A total of 1418 childhood ALL survivors were eligible in the 58LAE project, among whom 507 answered to the socio-economic questionnaire and were eligible for the QoL evaluation (see earlier publication [30]). Among them, 183 responded (36.1%) and 3 additional patients provided QoL data but no socio-economic data, leading to a total of 186 respondents (109 females and 77 males). Among the patients who did not participate (n = 911), 729 were due to lost to follow-up and 182 were due to refusal of participation. Disease characteristics were similar between the 1418 participants eligible for the 58LAE study and the 507 participants eligible for the QoL evaluation. Disease characteristics were also similar between participants who were assessed for QoL versus those not assessed for QoL (Table 1), except slightly more females (55%) among participants. Table S2 demonstrates descriptive statistics of the outcome scales for the complete cohort. The distribution of predictors of interest by covariates used for the adjusted models is presented in Table S3. Below we focus on results from the multivariable analyses (see Table 2, Table 3 and Table 4). Univariate statistics can additionally be found in the tables (Tables S4–S6).

### 3.2. Gender

Regarding the SF-12, no differences were found in MCS scores between females and males MCS (−1.86 points, *p* = 0.24) and PCS (−1.83 points, *p* = 0.11) scores than males (see Table 2), albeit these effects were insignificant. 

For the IOC-CS negative impact scales, females demonstrate a larger (negative) impact of cancer than males on life challenges (0.29 points, *p* = 0.02). 

Regarding the IOC-CS positive impact scales, body and health was less positively impacted in females, which was insignificant in the adjusted model (−0.25 points, *p* = 0.06), but was significant in the unadjusted model. In contrast, personal growth (0.18 points, *p* = 0.36) and socializing (0.10 points, *p* = 0.492) were more (positively) impacted in females. 

### 3.3. Age at Diagnosis

Based on the SF-12, patients <6 years at diagnosis have worse age at diagnosis had no impact on MCS scores (−0.11 points, *p* = 0.95) (see Table 3).

Based on the IOC-CS, a larger negative impact of cancer is observed in patients <6 years at diagnosis on the life challenges (0.30 points, *p* = 0.03). For the positive impact scales body and health domain (−0.20 points, *p* = 0.23), personal growth (−0.38 points, *p* = 0.08) and socializing (−0.50 points, *p* = 0.001) domains, patients <6 years at diagnosis were less positively impacted by cancer. Although there was an insignificant effect of age at diagnosis on personal growth in the adjusted model, this association was significant in the unadjusted model. 

### 3.4. Relapse/SN

Regarding the SF-12, patients with relapse/SN show worse MCS (−3.09 points, *p* = 0.13) and PCS scores (−4.0 points, *p* = 0.01) (see Table 4).

Based on the IOC-CS, patients with relapse/SN show a larger negative impact of cancer on life challenges (0.42 points, *p* = 0.01) than non-relapsed patients. These patients also show a less positive impact on body and health (−0.50 points, *p* = 0.004) and on socializing (−0.46 points, *p* = 0.01), whereas personal growth (0.75 points, *p* = 0.01) was more positively impacted in patients with relapse/SN. 

### 3.5. NCI Risk Group 

NCI high-risk patients show significantly worse (lower) PCS scores (−3.04 points, *p* = 0.01) compared to patients with NCI standard risk (see Table S7). Although they have higher MCS scores, this group difference was statistically insignificant (1.37 points, *p* = 0.43).

### 3.6. CRT 

Results suggest irradiated patients have worse PCS scores (−7.67 points, *p* = 0.001) (see Table S8). In addition, these patients show a larger negative impact on the IOC-CS thinking and memory problem scale, compared to patients with no CRT (0.32 points, *p* = 0.28). Although this effect was insignificant in the adjusted model, it was significant in the unadjusted model. In addition, the effect of CRT on PCS and thinking and memory problems did not depend on age at diagnosis, as the interaction term was insignificant.

## 4. Discussion

In this novel cohort study, we observed positive as well as adverse long-term outcomes in adult survivors of childhood ALL, depending on their patient and treatment characteristics including gender, age at diagnosis, relapse, CRT, and risk group. More specifically, younger (<6 years old), female, and relapsed patients might encounter more life challenges after their disease. Regarding physical outcomes, physical component scores were lower for relapsed, irradiated, and high-risk patients.

On the other hand, beneficial effects were encountered as well. Non-relapsed patients showed a more positive impact on body and health and socializing, while personal growth was more positively impacted in relapsed patients. Furthermore, older patients (>6 years) also demonstrated a more positive impact on their social experiences (i.e., socializing scale) than younger patients. As we investigated survivors in the adult stage of life, this study sheds new light on the long-term impact of certain disease-, treatment- and patient-related risk factors. 

### 4.1. Physical Sequelae

Regarding the SF-12 physical component, relapsed, higher risk and irradiated patients scored worse compared to their counterparts. First, relapsed patients have experienced a larger cancer burden and cumulative dosages of CRT and/or systemic chemotherapy. 

Given that physical functioning of the patients can be affected by both the disease as well as each of these treatment modalities (through osteonecrosis, loss of muscle strength, peripheral neuropathy, toxic processes to the central nervous system, pain, and fatigue [37]), both factors could explain the higher risk of physical problems in relapsed and irradiated subgroups. 

The significant adverse effect of irradiation on physical functioning, supports the successful replacement of cranial irradiation by chemotherapeutic regimens in more recent treatment protocols [31,38]. 

Not only physical functioning was lower in relapsed patients (as measured with the SF-12), but also their personal perspective on it after a cancer treatment was less positively influenced than in non-relapsed patients (as measured with the body and health scale of the IOC-CS). Survivors of childhood cancer in general might show lower levels of physical activity and increased risky health behavior [39]. Patients who relapsed or experienced a SN, might be physically challenged even more than non-relapsed patients, as they receive more treatments with physical side effects. These challenges could lead to body image dissatisfaction and decreased self-confidence [40] as well as a decrease in motivation or engagement for a healthy lifestyle, possibly including a less healthy diet. This could become a vicious circle, with a less healthy lifestyle increasing the risk of (second) cancer development [41] through multiple direct and indirect biochemical mechanisms. 

### 4.2. Mental and Psychosocial Sequelae 

Previous research has demonstrated that the majority of childhood cancer survivors are psychologically functioning well, but certain patients are at increased risk of having long-term difficulties [42]. Patient characteristics playing an important role at diagnosis and throughout development remains uncertain. By contrast, based on the IOC-CS, subgroup differences in personal growth experience, life challenges, and socializing were encountered. This suggests that the experienced impact of cancer depends on specific individual features. For instance, higher personal growth scores were encountered among ALL patients who relapsed. In other words, these patients consider having had cancer as a strengthening experience with positive effects on their life. This finding confirms previous findings of illness severity [43] and SN or relapse [44] as significant predictors of post-traumatic growth in children and adolescents surviving cancer. Barakat and colleagues (2006) also demonstrated that perception of the illness severity, rather than the objective illness severity itself, predicted the experience of post-traumatic growth [43]. Based on our findings, it can be assumed that relapsed patients experience their cancer as being more severe, possibly associated with more cancer-related worry and physical difficulties, whereas their mental functioning improves and they experience personal growth [45]. Although older and female patients also tended to show higher personal growth scores, which is in line with some earlier findings [46], these findings were not statistically significant in adjusted models. 

Such increases in personal growth as well as in independent living [19], might explain why some studies demonstrate improved QOL in survivors many years after treatment [22,28].

While relapsed patients might experience more personal growth (i.e., perceiving cancer as a part of own identity having a positive impact on life), they show higher scores in life challenges as well (i.e., emotional difficulties with having had the illness and being more afraid of it), which is consistent with earlier findings on increased risk for post-traumatic symptoms after relapse [47]. The fact that relapsed patients show both a higher positive impact as well as more challenges incorporating the experience in their life perception, might demonstrate the larger mental impact and proportion of the disease in these patients’ lives in general. The disease is clearly a complex experience that can both involve negative and positive consequences. The negative impact, on the scale of life challenges, might specifically require attention for clinical support and interventions in these patients.

Besides relapse, being younger (<6 years) at diagnosis and female appears to be potential additional risk factors leading to more life challenges (i.e., emotional problems with the disease), even after a long interval after their disease. Regarding the younger age, the large traumatic experience at young age might have increased the long-term disease-related anxiety (measured with the IOC-CS life challenges scale), even though such traumatic symptoms are by contrast not always observed by parents shortly after treatment [48]. Such age effects on disease-related anxiety in long-term survivors were not consistently reported in previous studies, and were mostly investigated in adolescents [45]. Therefore, the elevated risk of younger age at diagnosis for the life challenges scale needs further investigation. The higher scores of life challenges in females (i.e., disease-related worry) replicates previous findings [45].

Worrying about the disease and its impact could be challenging for specific ALL patients. Some might face more socializing adjustment difficulties [39]. More specifically, we encountered that older (≥6 years) and non-relapsed patients in our cohort demonstrated a more positive impact on socializing than younger and relapsed patients, respectively. Regarding this age effect, a possible interpretation is that adolescence is featured with strong transition from parental input to the larger influence of peer relationships [49]. Thus, a diagnosis of ALL during adolescence, could positively affect this social transition phase in some of these emerging adults [50]. In addition, relationships of adolescents are differently defined compared to children. In adolescents, friendship includes more in-depth and personal conversations, which could largely be influenced during a cancer experience [51,52,53]. Although a more positive impact was found on socializing and less life challenges in children ≥6 years than younger peers, the number of adolescents in our population was only small. Furthermore, other studies have shown that adolescents could also be more vulnerable for specific social outcomes (e.g., family concerns) after treatment [54]. Hence, future studies are recommended investigating larger adolescent sample sizes to explore these psychosocial developmental stages in more detail. 

### 4.3. Limitations 

The QoL scales and their corresponding predictor sets were predefined based on the literature and clinical expertise. Still, we cannot exclude the possibility that certain associations with other potential risk factors (e.g., chemo- or radiotherapy doses, family size, and support) that were not investigated might have been missed. In addition, although some investigated associations were statistically significant (according to the set 5% significance level), there are still gaps in the literature with respect to whether the observed effect sizes are also clinically relevant. 

Second, this study focused on long-term survivors of childhood ALL, with a survivorship range between 12.9 and 41.6 years. Thus, only conclusions could be inferred for the long-term survivors, while the impact of cancer and its treatment could be different in shorter terms. Given that the age range of participants in this study was relatively large (i.e., 18.1–52.8 years), assessments took place at different physical and mental stages of life of participants. Furthermore, although the distributions of clinical characteristics (treatment-, and disease-related features) were similar between respondents and non-respondents, we recognize that a selection bias cannot be excluded (given that not all 510 participants of the original database participated in the current QoL study). Furthermore, given the very long-term follow-up (investigations took place in adulthood), the participation was limited to 37%. This participation rate however is in line with earlier long-term QoL survivorship studies in childhood cancer populations [55]. Of note, there were slightly more females in the subgroup of respondents and 55% of the survivors had a high level of education. Nevertheless, we wish to stress the fact that this study is unique in investigating the daily life experiences of long-term adult survivors of childhood ALL, as they are confronted with new life challenges. 

Third, the IOC-CS was validated for childhood cancer survivors, but only limitedly investigated and implemented so far. Hence, comparisons between the results of this study and previous findings regarding the IOC-CS remain limited. Finally, all survivors were European participants, with all data acquired in France and Belgium, so the possibility of cultural-specific findings cannot be excluded. Given that the focus of this paper was to compare risk groups within the patient population, we would recommend future research to elaborate on group comparisons between patients versus a normative data. 

### 4.4. Clinical Implications and Future Directions

This is a novel study reporting individual risk factors for QoL outcomes in the very long-term. Although in general, the survivors have scores within the normal range on the measured QoL scales, younger, female, and relapsed patients might still be more vulnerable for some psychological aspects than their counterparts. Hence, these patients might need more in-depth psychological follow-up. Physical challenges by contrast could specifically be the case for relapsed and high-risk patients. For this subgroup, adapted rehabilitative support and information on healthy nutrition and physical exercise (e.g., by a dietician and physiotherapist, resp.) might result in better physical QoL outcomes. Finally, psychological support for mental health, education, as well as sufficient physical activity and living a healthy life can be beneficial for all childhood leukemia patients in the longer run. Furthermore, individualized support should be foreseen for all long-term survivors in specialized late effect clinics. Future studies are needed to investigate the efficacy of such individually tailored interventional trials, with interventions planned as early onwards as possible. Moreover, this study focused on the demographic and clinical risk factors for QoL-related outcomes. However, (pre-existing) patient-, parent-, and family-specific features, such as personality, coping mechanisms [56], sleep [29], and neurocognitive functioning [57], could additionally contribute to the daily life quality of survivors, which will need further investigation for their partial effects. 

## 5. Conclusions

Based on this adult cohort survivor study, we conclude beneficial as well as adverse physical and psychological self-reported outcomes in the long run, after childhood ALL. Given that younger, female, and relapsed patients might experience more life challenges up to many years after treatment, psychosocial support focusing on this issue could be recommended. On the other hand, relapse, irradiation, and high-risk categorization might lead to more physical challenges, which should receive specialized physiotherapeutic interventions, including support of a healthier lifestyle. 

## Figures and Tables

**Table 1:** Demographic and clinical characteristics of childhood ALL survivors.

	QoL Assessed		Total (N = 510)
	No (N = 324)	Yes (N = 186)	
	N (%)	N (%)	N (%)
**EORTC protocol**			
58741	8 (2.5)	17 (9.1)	25 (4.9)
58831/2	67 (20.7)	43 (23.1)	110 (21.6)
58881	249 (76.9)	126 (67.7)	375 (73.5)
**Gender**			
Male	154 (47.5)	77 (41.4)	231 (45.3)
Female	170 (52.5)	109 (58.6)	279 (54.7)
**Age at diagnosis (years)**			
<6	220 (67.9)	115 (61.8)	335 (65.7)
≥6	104 (32.1)	71 (38.2)	175 (34.3)
Range	0.3–17.9	0.2–14.7	0.2–17.9
Mean (SD)	5.43 (3.56)	5.62 (3.30)	5.50 (3.47)
**Age at follow-up (years)**			
<18–24	158 (48.8)	85 (45.7)	243 (47.6)
≥25	166 (51.2)	101 (54.3)	267 (52.4)
Range	18.1–51.1	18.1–52.8	18.1–52.8
Mean (SD)	25.98 (5.60)	27.61 (7.09)	26.57 (6.23)
**Country**			
Belgium	89 (27.5)	148 (79.6)	237 (46.5)
France	235 (72.5)	38 (20.4)	273 (53.5)
**WBC at diagnosis (×10^9^/L)**			
<25	239 (73.8)	125 (67.2)	364 (71.4)
≥25	85 (26.2)	61 (32.8)	146 (28.6)
**NCI risk group**			
Standard Risk	230 (71.0)	131 (70.4)	361 (70.8)
High Risk	94 (29.0)	55 (29.6)	149 (29.2)
**HSCT**			
No	298 (92.0)	174 (93.5)	472 (92.5)
Yes	26 (8.0)	12 (6.5)	38 (7.5)
**Relapse**			
No	282 (87.0)	161 (86.6)	443 (86.9)
Yes	42 (13.0)	25 (13.4)	67 (13.1)
**Relapse or SN**			
No	274 (84.6)	151 (81.2)	425 (83.3)
Yes	50 (15.4)	35 (18.8)	85 (16.7)
**CRT**			
No	292 (90.1)	157 (84.4)	449 (88.0)
Yes	32 (9.9)	29 (15.6)	61 (12.0)
**Endocrine disorders**			
No	74 (22.8)	36 (19.4)	110 (21.6)
Yes	45 (13.9)	24 (12.9)	69 (13.5)
Missing	205 (63.3)	126 (67.7)	331 (64.9)
**Being married or living with a partner**			
No	200 (61.7)	112 (60.2)	312 (61.2)
Yes	122 (37.7)	69 (37.1)	191 (37.5)
Missing	2 (0.6)	5 (2.7)	7 (1.4)
**University degree**			
No	155 (47.8)	85 (45.7)	240 (47.1)
Yes	164 (50.6)	95 (51.1)	259 (50.8)
Missing	5 (1.5)	6 (3.2)	11 (2.2)
**Currently working**			
No	124 (38.3)	57 (30.6)	181 (35.5)
Yes	197 (60.8)	125 (67.2)	322 (63.1)
Missing	3 (0.9)	4 (2.2)	7 (1.4)

Note: ALL = acute lymphoblastic leukemia, CRT = cranial radiotherapy, HSCT = hematopoietic stem cell transplantation, NCI = National Cancer Institute, SD = standard deviation, SN = second neoplasm, WBC = white blood cells. EORTC 58741 (1971–1978), 58831/2 (1983–1989), 58881 (1989–1998).

**Table 2:** Associations between gender and SF-12 subscales and IOC-CS subscales based on a multivariable regression analysis.

	SF-12				IOC-CS							
	Mental Component Summary		Physical Component Summary		Positive Impact Subscale						Negative Impact Subscale	
					Body and Health (+)		Personal Growth (+)		Socializing (+)		Life Challenges (-)	
Parameter	Estimate (SE)	*p*-Value	Estimate (SE)	*p*-Value	Estimate (SE)	*p*-Value	Estimate (SE)	*p*-Value	Estimate (SE)	*p*-Value	Estimate (SE)	*p*-Value
Intercept	49.85 (1.83)	<0.0001	57.0 (1.31)	<0.0001	3.63 (0.15)	<0.0001	3.14 (0.23)	<0.0001	4.22 (0.16)	<0.0001	1.68 (0.14)	<0.0001
Gender (ref: Male)	−1.86 (1.56)	0.24	−1.83 (1.12)	0.11	−0.25 (0.13)	0.06	0.18 (0.19)	0.36	0.10 (0.14)	0.49	0.29 (0.12)	**0.02**
Age at follow-up (Ref: ≥25)	−2.56 (1.95)	0.19	−0.11 (1.40)	0.94	0.16 (0.16)	0.33	−0.28 (0.24)	0.24	0.15 (0.17)	0.39	0.03 (0.15)	0.84
Protocol (ref = 58881) 58741	0.13 (2.96)	0.97	−11.65(2.12)	<0.0001	−0.27 (0.26)	0.30	−0.40 (0.38)	0.29	−0.62 (0.27)	**0.02**	0.36 (0.23)	0.13
58831/2	−1.50 (2.30)	0.52	−1.15 (1.66)	0.49	0.01 (0.18)	0.94	−0.31 (0.27)	0.26	−0.31 (0.19)	0.11	0.19 (0.17)	0.28

Note: (+) is indicated for positive impact scales of the IOC-CS, (-) is indicated for negative impact scales of the IOC-CS. For positive impact scales, a higher mean score indicates a larger positive impact, while for the negative impact scales, a higher mean score indicates a larger negative impact. EORTC 58741 (1971–1978), 58831/2 (1983–1989), 58881 (1989–1998).

**Table 3:** Associations between age at diagnosis and the SF-12 mental subscale and IOC-CS subscales based on a multivariable regression analysis.

	SF-12		IOC-CS							
	Mental Component Summary		Positive Impact Subscale						Negative Impact Subscale	
			Body and Health (+)		Personal Growth (+)		Socializing (+)		Life Challenges (-)	
Parameter	Estimate (SE)	*p*-Value	Estimate (SE)	*p*-Value	Estimate (SE)	*p*-Value	Estimate (SE)	*p*-Value	Estimate (SE)	*p*-Value
Intercept	49.87 (1.86)	<0.0001	3.65 (0.15)	<0.0001	3.19 (0.23)	<0.0001	4.28 (0.16)	<0.0001	1.64 (0.14)	<0.0001
Age at diagnosis (Ref: ≥6)	−0.11 (1.88)	0.95	−0.20 (0.15)	0.23	−0.38 (0.22)	0.08	−0.50 (0.15)	**0.001**	0.30 (0.14)	**0.03**
Gender (ref: Male)	−1.85 (1.57)	0.24	−0.22 (0.13)	0.10	0.22 (0.20)	0.25	0.16 (0.13)	0.25	0.26 (0.12)	**0.03**
Age at follow-up (Ref: ≥25)	−2.49 (2.32)	0.28	0.27 (0.19)	0.15	−0.04(0.27)	0.89	0.46 (0.19)	**0.02**	−0.16 (0.17)	0.35
Protocol (ref = 58881) 58741	0.16 (3.02)	0.96	−0.21 (0.26)	0.43	−0.27 (0.38)	0.48	−0.46 (0.27)	0.10	0.27 (0.26)	0.25
58831/2	−1.45 (2.43)	0.55	0.10 (0.19)	0.68	−0.17 (0.28)	0.55	−0.13 (0.20)	0.50	0.08 (0.18)	0.70

Note: (+) is indicated for positive impact scales of the IOC-CS, (-) is indicated for negative impact scales of the IOC-CS. For positive impact scales, a higher mean score indicates a larger positive impact, while for the negative impact scales, a higher mean score indicates a larger negative impact. EORTC 58741 (1971–1978), 58831/2 (1983–1989), 58881 (1989–1998).

**Table 4:** Associations between relapse/second cancer status and SF-12 subscales and IOC-CS subscales based on a multivariable regression analysis.

	SF-12				IOC-CS							
	Mental Component Summary		Physical Component Summary		Positive Impact Subscale						Negative Impact Subscale	
					Body and Health (+)		Personal Growth (+)		Socializing (+)		Life Challenges (-)	
Parameter	Estimate (SE)	*p*-Value	Estimate (SE)	*p*-Value	Estimate (SE)	*p*-Value	Estimate (SE)	*p*-Value	Estimate (SE)	*p*-Value	Estimate (SE)	*p*-Value
Intercept	50.29(1.84)	<0.0001	57.58(1.30)	<0.0001	3.69 (0.15)	<0.0001	3.06 (0.22)	<0.0001	4.28 (0.16)	<0.0001	1.63 (0.14)	<0.0001
Relapse or second cancer (Ref = No)	−3.09 (2.0)	0.13	−4.06(1.43)	**0.01**	−0.50(0.17)	**0.004**	0.71 (0.25)	**0.01**	−0.46 (0.18)	**0.01**	0.42 (0.16)	**0.01**
Gender (ref: Male)	−1.78 (1.56)	0.25	−1.75(1.10)	0.11	−0.21(0.13)	**0.10**	0.13(0.19)	**0.50**	0.13(0.14)	**0.35**	0.26 (0.12)	**0.03**
Age at follow-up (Ref: ≥25)	−2.64 (1.95)	0.18	−0.23(1.37)	0.87	0.12(0.16)	0.45	−0.22(0.23)	0.34	0.11(0.17)	0.50	0.06 (0.15)	0.67
Protocol (ref = 58881) 58741	0.42 (2.96)	0.89	−11.26(2.08)	**<0.0001**	−0.17(0.25)	0.50	−0.54(0.38)	0.15	−0.54(0.27)	0.05	0.29 (0.23)	0.20
58831/2	−1.06 (2.31)	0.65	−0.65(1.63)	0.69	0.05(0.18)	0.77	−0.36(0.27)	0.18	−0.28(0.19)	0.14	0.16 (0.17)	0.36

Note: (+) is indicated for positive impact scales of the IOC-CS, (-) is indicated for negative impact scales of the IOC-CS. For positive impact scales, a higher mean score indicates a larger positive impact, while for the negative impact scales, a higher mean score indicates a larger negative impact. EORTC 58741 (1971–1978), 58831/2 (1983–1989), 58881 (1989–1998).

## Data Availability

Data can become available after an official data sharing request, internal review and approval by the EORTC.

## Supplementary Materials

The following supporting information can be downloaded at: https://www.mdpi.com/article/10.3390/cancers14010152/s1, Supplementary Figure S1: Treatment protocols in detail; Supplementary Table S1: Summary of predictors of interest, QoL outcome subscales and covariates used for the adjusted models. Supplementary Table S2: Descriptive statistics SF-12 and IOC-CS. Supplementary Table S3: Distribution of predictors of interest by covariates. Supplementary Tables S4–S8: Univariate and additional (NCI risk and CRT) analyses.
