# Supplementary material for: Sociodemographic and Medical Determinants of Quality of Life in Long-Term Childhood Acute Lymphoblastic Leukemia Survivors Enrolled in EORTC CLG Studies
Source: Cancers (Basel). 2021 Dec 29;14(1):152. doi: 10.3390/cancers14010152 (PMC8750449; doi:10.3390/cancers14010152)
Supplement: Supplementary file 1 [file cancers-14-00152-s001.zip › cancers-1493413-supplementary.pdf]

Figure S1: Treatment protocols in detail

EORTC study 58741

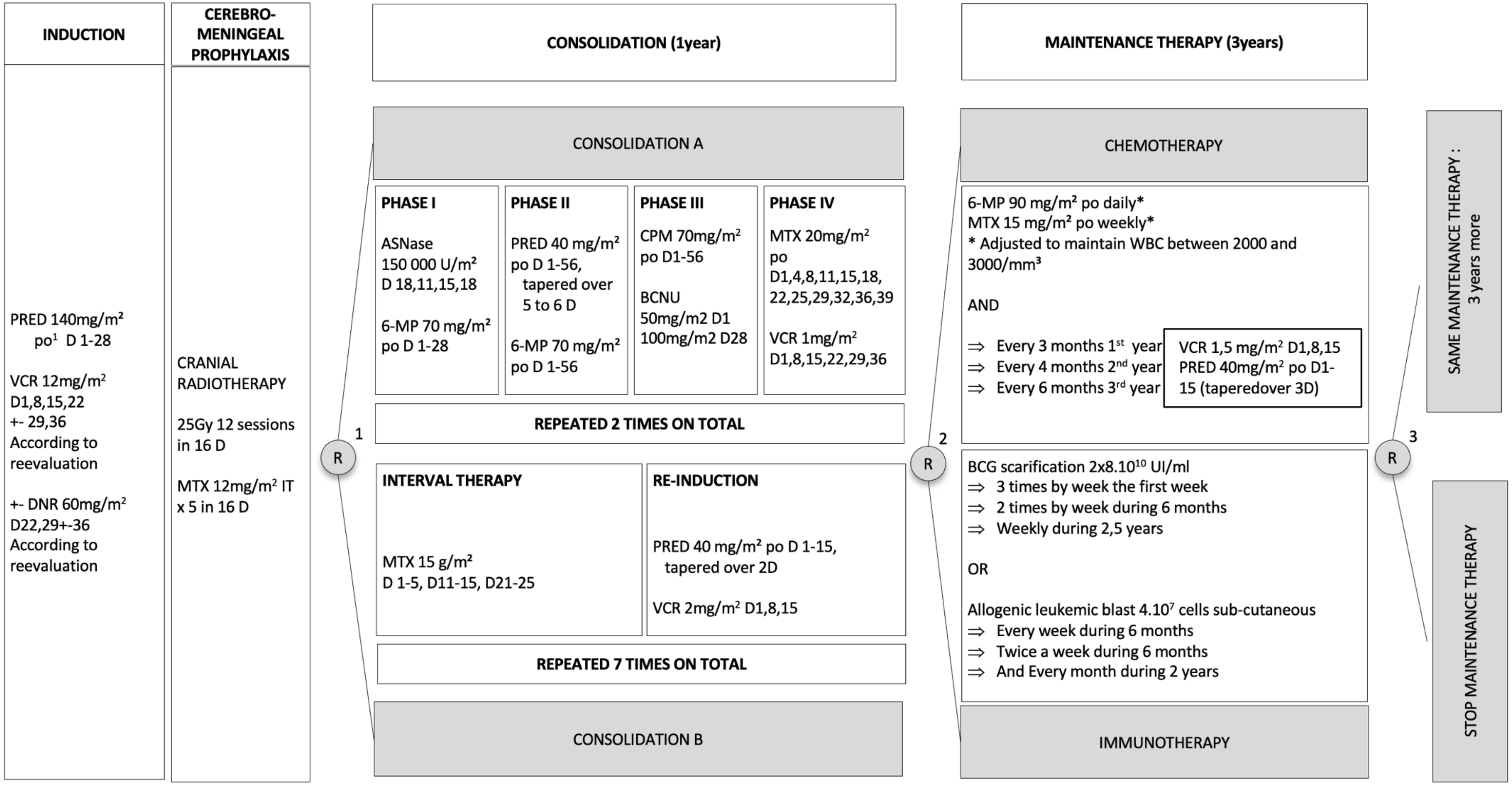

Notes:

[1] per oral (when not specified, administration is intravenous)

Abbreviations: ASNase: asparaginase, BCNU : Carmustine, CPM: cyclophosphamide, DNR: daunorubicin, 6-MP: 6-mercaptopurine, MTX: methotrexate, PRED: prednisone, R: randomization, VCR: vincristine

EORTC study 58831: standard risk patients (RF<1.2) in CR at the end of induction

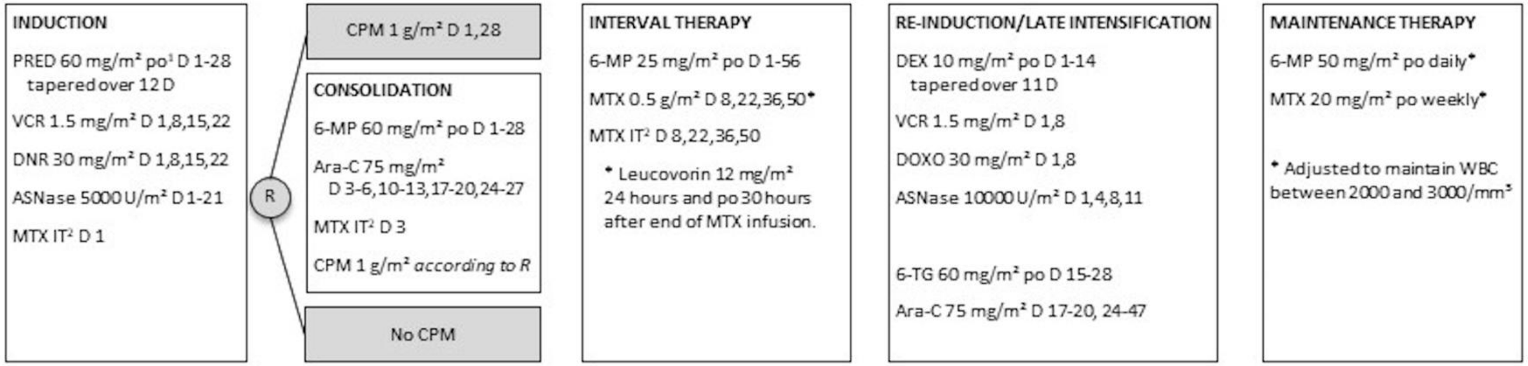

EORTC study 58832: medium risk patients (RF 1.2-1.69), high risk patients (RF≥1.7), and standard risk patients (RF<1.2) not in CR at the end of induction

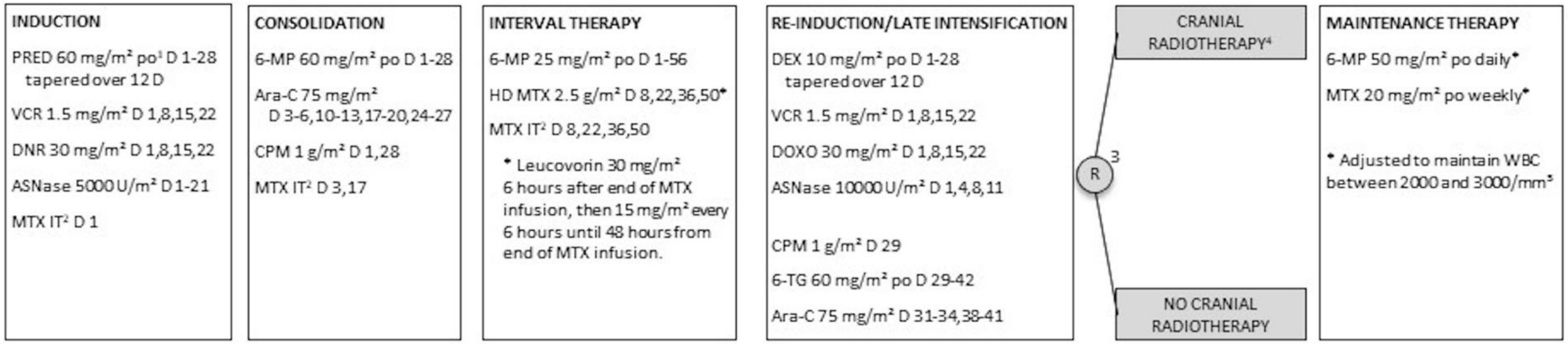

Notes:

[1] Per oral (when not specified, administration is intravenous)

[2] Intrathecal at the dose of 6 mg < 1 year, 8 mg 1-< 2 year, 10 mg 2-<3 year, 12 mg ≥ 3 year

[3] Patients in complete remission at the end of consolidation and still in complete remission after the end of late intensification

[4] Dose according to age : 16 Gy (if age < 1 year), 20 Gy (if age 1-< 2 years) or 24 Gy (if age ≥ 2 years)

Abbreviations: Ara-C: cytarabine, ASNase: asparaginase, CPM: cyclophosphamide, CR: complete remission, D: day, DEX: dexamethasone, DNR: daunorubicin, DOXO: doxorubicin, HD: high-dose, IT: intrathecal, 6-MP: 6-mercaptopurine, MTX: methotrexate, PRED: prednisone, R: randomization, 6-TG: 6-thioguanine, VCR: vincristine, WBC: white blood cells

Table S1: Summary of predictors of interest, QoL outcome subscales and covariates used for the adjusted models.

Predictors of QoL of interest	QoL outcome	Covariates for adjusted model
Gender	• SF-12: Mental and Physical component summary<br>• IOC-CS: Life challenges, Personal growth, Socializing and Body and health	Protocol, Age at follow-up
Age at diagnosis	• SF-12: Mental component summary<br>• IOC-CS: Life challenges, Personal growth, Socializing and Body and health	Protocol, Age at follow-up, Gender
Relapse/SN	• SF-12: Mental and Physical component summary<br>• IOC-CS: Life challenges, Personal growth, Socializing and Body and health	Protocol, Age at follow-up, Gender
NCI risk group	• SF-12: Mental and Physical component summary	Protocol, Age at follow-up, Gender
CRT and its interaction with age at diagnosis	• SF-12: Physical component summary<br>• IOC-CS : Thinking/memory problems	Age at diagnosis, Age at follow-up, Gender

Abbreviations: CRT: Cranial Radiotherapy, IOC-CS: Impact of Cancer-Childhood Survivors, NCI: National Cancer Institute, QoL: quality of life, SF-12: Short-Form Health Survey, SN: second neoplasm

Table S2: Descriptive summary of the SF-12 and IOC-CS scales used

	**Total (N=186)**
SF-12	
Mental component summary	
Median	49.5
Range	18.4 - 62.5
Mean (SD)	47.25 (9.97)
N	171
Physical component summary	
Median	57.1
Range	24.7 - 67.0
Mean (SD)	54.52 (7.87)
N	170
IOC-CS	
Life challenges (-)	
Median	1.9
Range	1.0 - 4.6
Mean (SD)	1.95 (0.70)
N	141
Personal growth (+)	
Median	3.1
Range	1.0 - 5.0
Mean (SD)	3.02 (1.08)
N	138
Socializing (+)	
Median	4.7
Range	1.0 - 5.0
Mean (SD)	4.23 (0.80)
N	139
Thinking and memory problems (-)	
Median	2.0
Range	1.0 - 4.4
Mean (SD)	2.24 (0.83)
N	141
Body and health (+)	
Median	3.6
Range	1.4 - 5.0
Mean (SD)	3.53 (0.75)
N	140

Abbreviations: IOC-CS: Impact of Cancer-Childhood Survivors, SF-12: Short-Form Health Survey.
Note. (+) is indicated for positive impact scales of the IOC-CS, (-) is indicated for negative impact scales of the IOC-CS. For positive impact scales, a higher mean score indicates a larger positive impact, while for the negative impact scales, a higher mean score indicates a larger negative impact.

e S3: Distribution of predictors of interest by covariates used for the adjusted models

	Age at diagnosis, years		Sex		Relapse or Second cancer		NCI risk group		Cranial radiotherap	
	<6 (N=115)	>=6 (N=71)	Male (N=77)	Female (N=109)	No (N=151)	Yes (N=35)	Standard Risk (N=131)	High Risk (N=55)	No (N=157)	Yes (N=29)
	N (%)	N (%)	N (%)	N (%)	N (%)	N (%)	N (%)	N (%)	N (%)	N (%)
**Male**	45 (39.1)	32 (45.1)			64 (42.4)	13 (37.1)	55 (42.0)	22 (40.0)	66 (42.0)	11 (37.9
**Female**	70 (60.9)	39 (54.9)			87 (57.6)	22 (62.9)	76 (58.0)	33 (60.0)	91 (58.0)	18 (62.1
**t diagnosis, years**										
**<6**			45 (58.4)	70 (64.2)	97 (64.2)	18 (51.4)	91 (69.5)	24 (43.6)	99 (63.1)	16 (55.2
**>=6**			32 (41.6)	39 (35.8)	54 (35.8)	17 (48.6)	40 (30.5)	31 (56.4)	58 (36.9)	13 (44.8
**t follow-up, years**										
**<18-24**	72 (62.6)	13 (18.3)	37 (48.1)	48 (44.0)	75 (49.7)	10 (28.6)	67 (51.1)	18 (32.7)	82 (52.2)	3 (10.3
**>=25**	43 (37.4)	58 (81.7)	40 (51.9)	61 (56.0)	76 (50.3)	25 (71.4)	64 (48.9)	37 (67.3)	75 (47.8)	26 (89.7
**col**										
**58741**	9 (7.8)	8 (11.3)	7 (9.1)	10 (9.2)	12 (7.9)	5 (14.3)	11 (8.4)	6 (10.9)	0 (0.0)	17 (58.6
**58831**	26 (22.6)	17 (23.9)	15 (19.5)	28 (25.7)	30 (19.9)	13 (37.1)	30 (22.9)	13 (23.6)	35 (22.3)	8 (27.6
**58881**	80 (69.6)	46 (64.8)	55 (71.4)	71 (65.1)	109 (72.2)	17 (48.6)	90 (68.7)	36 (65.5)	122 (77.7)	4 (13.8

Table S4: Univariate associations between gender and SF-12 subscales and IOC-CS subscales based on a univariate analysis

	SF-12				***IOC-CS***				
					Positive impact subscale				
	Mental component summary		***Physical component summary***		***Body and health (+)***		***Personal growth (+)***		
Parameter	Estimate (SE)	P-value	Estimate (SE)	P-value	*Estimate (SE)*	*P-value*	*Estimate (SE)*	*P-value*	*Esti (S*
Intercept	48.34 (1.20)	<.001	55.73 (0.94)	<.0.00 1	3.70 (0.10)	<.0001	2.92 (0.15)	<.0001	4.22
Gender (ref: Male)	-1.83 (1.55)	0.24	-2.04 (1.22)	0.10	-0.27 (0.13)	**0.04**	0.16 (0.19)	0.40	0.02

Note. (+) is indicated for positive impact scales of the IOC-CS, (-) is indicated for negative impact scales of the IOC-CS. For positive impact scales, a higher mean score indicates a larger positive impact, while for the negative impact scales, a higher mean score indicates a larger negative impact.

Table S5: Univariate associations between age at diagnosis and the SF-12 mental subscale and IOC-CS subscales based on a univariate analysis

	SF-12		***IOC-CS***					
	Mental component summary		***Positive impact subscale***					
			Body and health (+)		***Personal growth (+)***		***Socializing***	
Parameter	Estimate (SE)	P-value	*Estimate (SE)*	*P-value*	*Estimate (SE)*	*P-value*	*Estimate (SE)*	*P*
Intercept	48.14 (1.25)	<.0001	3.58 (0.10)	<.0001	3.25 (0.14)	<.0001	4.39 (0.11)	
Age at diagnosis (Ref: >=6)	-1.42 (1.58)	0.37	-0.08 (0.13)	0.53	-0.37 (0.19)	0.05	-0.27 (0.14)	

Note. (+) is indicated for positive impact scales of the IOC-CS, (-) is indicated for negative impact scales of the IOC-CS. For positive impact scales, a higher mean score indicates a larger positive impact, while for the negative impact scales, a higher mean score indicates a larger negative impact.

Table S6: Univariate associations between relapse/second cancer status and SF-12 subscales and IOC-CS subscales based on a univariate analysis

	SF-12				***IOC-CS***				
					Positive impact subscale				
	Mental component summary		***Physical component summary***		***Body and health (+)***		***Personal growth (+)***		
Parameter	Estimate (SE)	P-value	Estimate (SE)	P-value	*Estimate (SE)*	*P-value*	*Estimate (SE)*	*P-value*	*Esti (S*
Intercept	47.78(0.84)	<.0001	55.36(0.65)	<.0001	3.62(0.07)	<.0001	2.92 (0.10)	<.0001	4.32

Relapse or second cancer (Ref=No)	-2.91 (1.97)	0.14	-4.75(1.55)	0.003	-0.58 (0.17)	**0.001**	0.67 (0.25)	**0.01**	-0.57

Note. (+) is indicated for positive impact scales of the IOC-CS, (-) is indicated for negative impact scales of the IOC-CS. For positive impact scales, a higher mean score indicates a larger positive impact, while for the negative impact scales, a higher mean score indicates a larger negative impact.

Table S7: Associations between NCI risk group versus mental component summary (MCS) and physical component summary (PCS) scores of the SF-12

Parameter	***Mental component summary***				***Physical component summary***			
	Univariate		*Multivariable*		*Univariate*		*Multivariable*	
	Estimate (SE)	*P-value*	*Estimate (SE)*	*P-value*	*Estimate (SE)*	*P-value*	*Estimate (SE)*	*P-value*
Intercept	46.73 (1.98)	<.0001	49.73 (2.51)	<.0001	58.75 (1.79)	<.0001	58.35 (1.40)	<.0001
NCI risk group (Ref= Standard)	1.76 (1.68)	0.29	1.37 (1.73)	0.43	-3.28 (1.32)	0.01	-3.04(1.22)	**0.01**
Gender (ref: Male)			-1.90 (1.57)	0.23			-1.75(1.10)	0.12
Age at follow-up (Ref: >=25)			-2.22 (2.0)	0.27			-0.86(1.41)	0.54
Protocol (ref=58881) 58741			0.25 (2.97)	0.93			-11.91(2.09)	**<.0001**
58831/2			-1.19 (2.33)	0.61			-1.89(1.72)	0.26

Note. (+) is indicated for positive impact scales of the IOC-CS, (-) is indicated for negative impact scales of the IOC-CS. For positive impact scales, a higher mean score indicates a larger positive impact, while for the negative impact scales, a higher mean score indicates a larger negative impact.

Table S8: Associations between CRT (interaction with age at diagnosis) versus PCS scores and TMP scores IOC-CS

	SF-12-Physical component summary				***IOC-CS-Thinking and memory problems (-)***			
	Univariate		*Multivariable*		*Univariate*		*Multivariable*	
Parameter	*Estimate (SE)*	*P-value*	*Estimate (SE)*	*P-value*	*Estimate (SE)*	*P-value*	*Estimate (SE)*	*P-value*
Intercept	55.80 (0.61)	<.0001	56.7 (1.22)	<.0001	2.18(0.07)	<.0001	2.11(0.15))	<.0001
CRT(Ref=No)	-8.13(1.53)	**<.0001**	-7.67(2.30)	**0.001**	0.44 (0.20)	**0.03**	0.32(0.29)	0.28
Age at diagnosis (Ref: >=6)			-0.87(2.83)	0.67			0.51(0.37)	0.17
CRT* Age at diagnosis			0.001(3.17)	0.81			0.20(0.41)	0.63
Gender (ref: Male)			-1.55(1.16)	0.13			-0.06(0.15)	0.69
Age at follow-up (Ref: >=25)			1.05(1.35)	0.90			-0.18(0.17)	0.30

Note. (+) is indicated for positive impact scales of the IOC-CS, (-) is indicated for negative impact scales of the IOC-CS. For positive impact scales, a higher mean score indicates a larger positive impact, while for the negative impact scales, a higher mean score indicates a larger negative impact.
